# Pennsylvania Hospital

**Published:** 1851-02

**Authors:** J. Aitken Meigs


*Pennsylvania Hospital. Medical Wards, service of* Dr. Wood.
*Reported by* Mr. J. Aitken Meigs.

Wednesday, January 8th.—The following cases were brought before the class by Dr. Wood.

Case I.—*Partial hemiplegia resulting from arteritis.*

The patient, a man of robust constitution, æt. about 50, came into the house a few days before, complaining of some difficulty of phonation. The tongue, when protruded, evinced a slight tendency to depart from the median line towards the right, and a close inspection of the countenance betrayed a marked inequality in the expression of the two sides. The right half manifested in repose a more decided degree of muscular relaxation than the left, an indication generally accompanying some form of paralysis. When told to move his right arm he did so with difficulty; and the motions of the right leg were also somewhat impaired. Here was evidently partial hemiplegia, traceable undoubtedly to some lesion or disturbance seated in the encephalon, probably on the left side.

Upon further examination, no pulsation whatever could be detected in the radial, brachial, or axillary arteries, either of the right or left arm. When the arm was compressed, however, the veins filled freely and naturally, showing clearly that the want of pulsation was not assignable to a complete obstruction of the arterial vessels. This abnormal condition was confined to the upper extremities entirely, as the arteries of the lower limbs were found to pulsate freely. The left antero-lateral portion of the neck was slightly swollen, and the venous tracts of this region considerably distended. Pulsation was manifest at the root of the carotid of this side, though none could be detected above. Over the right carotid, however, and under the sternal extremity of the right clavicle, the arterial pulse was strong and distinct.

It was evident that between this point and the axillary artery, some impediment must exist, sufficient to break the impulse of the heart, and prevent its being extended down the artery of the arm, but insufficient altogether to obstruct the flow of blood. This impediment was probably in the subclavian artery, as pulsation could be felt in the right carotid.

In like manner there was probably some impediment in the left subclavian, affecting also in some degree the left carotid, as no pulsation could be felt in the axilla, and only a slight one near the lower extremity of the carotid of that side.

That this impediment was not within the aorta was proved by the active pulsation of that vessel to be felt at the epigastrium, and also by the pulsation of the arteries in the lower extremities.

Percussion over the upper and anterior part of the chest, both over the sternum and on each side of it, was quite clear, showing that no tumor existed in that position, which could interfere with the circulation.

There was a slight roughness of the sounds of the heart when listened to, over that organ, but by no means very distinct. At the sternal extremity of the right clavicle, the double sound was even louder than over the heart, and was attended with a very striking musical tone.

The aneurismal thrill could not be detected.

The double sound could also be heard behind, between the upper portion of the scapulæ.

Upon a view of all the circumstances, Dr. Wood gave it as his opinion, that at some former period *arteritis* had existed, producing the effusion of coagulable lymph within the vessels; and that in this way the subclavians had become partially obstructed, so as altogether to prevent the impulse from the heart being carried into the arteries of the upper extremities.

Not impossibly there might be dilatation in the arch of the aorta, upon the left, or some undiscoverable tumour, pressing upon the veins which return the blood of the left side of the head, thus accounting for the obvious venous tumefaction upon that side, and in some degree explaining the hemiplegic symptoms by the consequent venous congestion of the brain. The deficient supply of blood to the brain through the left carotid might also, possibly, have something to do with these symptoms; as the obvious deficiency of power upon the right side of the body, might result either from pressure on the nervous centres, or want of the requisite power in them from deficiency of blood.

Upon these views as to the patholgy of the case under consideration, Dr. Wood based his treatment. The main indication was, clearly, to cause absorption of the coagulable lymph coating the interior of the vessels. To this end, the great promoters of absorption, Mercury and Iodine, would be exhibited in moderate doses. Pilul. Hydrarg. gr. i. and Liq. Iodini Comp. gtt. x. were prescribed, each four times per diem.

Case II.—A woman, aged 51, who had entered the House some weeks ago. She had been troubled with incessant vomiting, which had continued three months, causing extreme emaciation, and threatening ultimate death, from interference with the function of nutrition. She had all the symptoms of chronic gastritis; and from the extreme anxiety, and the peculiar color and expression of her countenance, when she first came under notice, suspicion had been entertained of the existence of some carcinomatous affection. The following precription—

℞. Argenti Nitratis, gr. ss.
Pulv. Opii, gr. 1-6th. M.

was immediately administered three times a day, with the happy effect of an entire relief of the symptoms. She had vomited but once since entering the house—a period of three or four weeks. Her appetite has returned, she could easily retain and freely digest her ordinary food, and was obviously gaining flesh.

Case III. The next case presented to the class by Dr. Wood, was an Italian, who had entered the house suffering from a continued pain in the epigastrium, and considerable cough. He had been sick four months; but from his inability to speak the English language, no information whatever could be obtained from him as to his previous condition. His legs were highly œdematous, pitting distinctly and deeply on pressure. When in the horizontal position a general dropsical effusion was apparent, and fluctuation of a liquid in the abdomen was made sensible in the usual way. The intestines distended with flatus, and floating on the top of the water, gave forth a resonant sound on percussion. Effusion had also taken place into the pericardium, as shown by the dulness on percusssion over the thorax. The effusion, however, was slight, as the sounds of the heart, instead of being feeble, as would otherwise have been the case, were natural in the degree of loudness.

Auscultation revealed a murmur of regurgitation during the first sound of the heart, and directly over the mitral valve. From this circumstance, Dr. Wood concluded that the mitral valve was diseased, and allowed the blood to regurgitate during the ventricular systole. In consequence of this condition of the heart, the circulation was here impeded, and to the congestion of the lungs and of the right side of the heart, and the general venous system thus induced, was undoubtedly assignable the general anasarca under which the patient was laboring. Over the posterior and inferior portion of the right lung, crepitation could be distinctly heard; not the fine crepitant rale of pneumonia, but a sub-crepitation caused probably by the congested state of the pulmonary vessels.

The sputa were bronchitic in character; the urine had not been examined.

The patient was put upon the use of potassæ bi-tartrat. ℥j., to be taken in small doses during the day; and pulv. scillæ grs. ij. every two hours; to be diminished if it occasioned nausea. Cups were directed to be applied to the back of the chest, over the seat of the sub-crepitation.

January 12. The patient walked into the room unsupported and presented himself again to the class. The dropsical effusion had disappeared, the sub-crepitant rale was much less distinct, and he was already convalescent from his secondary affections. The abnormal sounds of the heart, however, still remained.

